# New 3D measurements of large redwood trees for biomass and structure

**DOI:** 10.1038/s41598-020-73733-6

**Published:** 2020-10-15

**Authors:** Mathias Disney, Andrew Burt, Phil Wilkes, John Armston, Laura Duncanson

**Affiliations:** 1 UCL Geography, Gower Street, London, WC1E 6BT UK; 2 NERC National Centre for Earth Observation (NCEO), UCL, Gower Street, London, WC1E 6BT UK; 3 Department of Geographical Sciences, University of Maryland, College Park, 2181 Lefrak Hall, College Park, MD 20742 USA

**Keywords:** Ecology, Forest ecology, Forestry, Carbon cycle

## Abstract

Large trees are disproportionately important in terms of their above ground biomass (AGB) and carbon storage, as well as their wider impact on ecosystem structure. They are also very hard to measure and so tend to be underrepresented in measurements and models of AGB. We show the first detailed 3D terrestrial laser scanning (TLS) estimates of the volume and AGB of large coastal redwood *Sequoia sempervirens* trees from three sites in Northern California, representing some of the highest biomass ecosystems on Earth. Our TLS estimates agree to within 2% AGB with a species-specific model based on detailed manual crown mapping of 3D tree structure. However TLS-derived AGB was more than 30% higher compared to widely-used general (non species-specific) allometries. We derive an allometry from TLS that spans a much greater range of tree size than previous models and so is potentially better-suited for use with new Earth Observation data for these exceptionally high biomass areas. We suggest that where possible, TLS and crown mapping should be used to provide complementary, independent 3D structure measurements of these very large trees.

## Introduction

Some of the highest values of forest above ground biomass (AGB) ever reported, up to $5190\,\hbox { t ha}^{-1}$, are from groves of *Sequoia sempervirens* and *Sequoiadendron giganteum* in Northern California^1–3^. These trees are also the tallest trees on Earth, routinely exceeding 100 m^4–6^. Estimating the size and mass of such trees is an extremely difficult task and is very rarely, if ever, done directly via destructive harvest of complete trees, for a variety of reasons^7,8^. The most compelling of these is of course the undesirability of damaging such magnificent and ancient trees, very often in protected areas. Even if harvest is possible e.g. in conjunction with timber operations, it is still very challenging due to the sheer size of the trees and so is done typically for trees $< 50$ m height. Destructive harvesting also requires great care to minimise damage during felling, and then sub-sampling of component volumes and masses that are then extrapolated to the whole tree^9^. Even when larger trees have been sampled, the measurements have been affected by the crowns being destroyed during felling^10^.

The importance of big trees, (typically defined as $> 70$ cm diameter-at-breast height, DBH), is widely-recognised in terms of carbon storage, demographics and impact on their surrounding ecosystems^11–14^. Unfortunately the importance of big trees is in direct proportion to the difficulty of measuring them, particularly for AGB. As a result, AGB is estimated using indirect approaches, the most widely-used being calibrated empirical allometric models relating DBH, height H (if available) to volume and then, via wood density or specific gravity, to AGB^15^. These allometric relationships represent the assumption of the general scale-invariance of the often (but not always) strong correlations between DBH, H and AGB, and are calibrated against destructively harvested data^15,16^. However even these destructively-harvested calibration data are very often only sub-samples of full trees, extrapolated using estimates of form and shape, particularly for large trees. For the very largest trees there are virtually no destructive harvest measurements at all and so AGB is inferred from allometric extrapolations based on much smaller trees. A key assumption of allometric models is that provided scale-invariance holds, then the distribution of tree size in the calibration data is irrelevant. However if scale-invariance is not true, then predictions for under-represented trees, likely to be the largest ones^18^, will be biased^17^.

An additional challenge when measuring very large trees is that the particular growth and survival strategies that allow for such size and age may also lead to departure from allometric models based on smaller trees^7,18^. For example, it has been observed that trees with main trunks that are large compared to their crowns (relative to other, smaller trees) produce more wood annually^19^. Specific traits of *Sequoia sempervirens* include shade tolerance, fire and and disease resistance, and a high capacity for sprouting and trunk reiteration. These traits may also mean that they are not well-represented by generalised allometries^8^. Longevity also implies that older, larger trees have encountered very different histories of fire, disease and climate variation than their smaller neighbours, presumably leading not only to potentially different growth forms but also greater variance.

To overcome some of the limitations of DBH:H allometry when measuring very large trees, a second indirect approach is to use either ground-based measurements of standing trees^2^ or far more detailed tree-climbing measurements, referred to as ‘crown mapping’ (see^18–20^ for example). The latter approach can be used to quantify the size of different components of an individual tree in situ (trunk, major branches, crown etc.), as well as potentially providing core samples of wood tissue from the different components. This provides a much more detailed 3D structural picture of a particular individual tree, but requires skilled and potentially difficult climbing activities. In addition, various choices are needed as to what to measure, where and how, and then how to combine the resulting measurements in to a more complete map of 3D structure and volume^20^.

The uncertainties of any indirect estimate of AGB are compounded by the need for values of wood density to convert volume to mass. The importance of wood density as a key plant trait that in some senses integrates form and function^21,22^ have led to published databases of values. These have been widely-used in estimating AGB from plot measurements of DBH^22^. Given the long history of commercial forestry management of *Sequoia sempervirens*, values of wood density (and specific gravity) have been published for some time^23^. However, even with reliable values of wood density, uncertainty in allometric estimates of AGB will also be a function of the variability of density within an individual tree. This can vary substantially in the radial direction, with height and wood type (cambium, heartwood, sapwood, compression or tension tissue etc). In addition the fraction of bark can vary substantially in large *Sequioa sempervirens*, making up 18% of the total wood volume in some cases^24,25^.

A fundamental issue when applying indirect models of this sort to estimating AGB is that at no stage are the resulting estimates directly validated i.e. by harvest and weighing. This makes it all the more important (but also difficult) to quantify estimated uncertainties, particularly when moving to larger scales (see the discussion by^26^ of this problem).

When moving up in scale e.g. via Earth Observation (EO), either at the tree-scale using airborne lidar, or at larger scales using satellite estimates of canopy height, some of the allometric uncertainties relating to random errors in individual tree size, form and wood density may average out^27,28^. Satellite estimates of AGB are based on area-averaged canopy height estimates (typically weighted by stem basal area) rather than individual tree heights^30^, which again will tend to reduce errors arising from using allometries derived from individual tree measurements. However, the accuracy of these estimates relies more generally on conformity of the wider sample to the underlying allometric harvest data^17^. Ultimately, all EO estimates are based on field estimates, and in turn on the allometric models applied to tree measurements. Uncertainties and biases in individual tree estimates that result from allometric models therefore potentially propagate to uncertainties and biases in EO products. When considering the biomass of plots containing exceptionally large individual trees, uncertainties are likely to be compounded because of the importance of these trees in the overall biomass budget^13^. This has implications for wide-area estimates of AGB from EO, which typically rely on H-based allometry and estimates of stem density or basal area. This requires calibration of these allometries (potentially using H to estimate DBH and then AGB), as well as specific ground measurements to validate the resulting EO-derived estimates of AGB, something that is currently very rarely done^29,30^.

One way to at least partially address the problem of validating indirect estimates, is by comparing methods that are independent, having totally different underlying assumptions, strengths and weaknesses. Terrestrial laser scanning (TLS) offers one such alternative, by providing estimates of detailed 3D tree structure and volume in even tall, dense forests^31,32^. While TLS estimates of tree volume (and AGB) are also indirect, they do not rely on the assumptions of allometric models. TLS estimates of volume and AGB have also been shown to agree closely with destructive harvest measurements of full trees^33^ as well as with harvested sub-components of larger tropical trees^34,35^. As a non-destructive technique, TLS data can also be collected in established forest plots, making them particularly suited for EO calibration and validation activities^36,37^.

Here, we aim to test whether TLS can provide accurate estimates of volume and hence AGB of large *Sequioa sempervirens* trees. We compare TLS measurements to an independent 3D crown-mapping approach, and to both species-specific and generalised allometric estimates of volume and AGB^8,9,19^. We develop a new allometric relationship spanning a far greater size-range of trees than currently possible.

## Results

**Figure 1:**
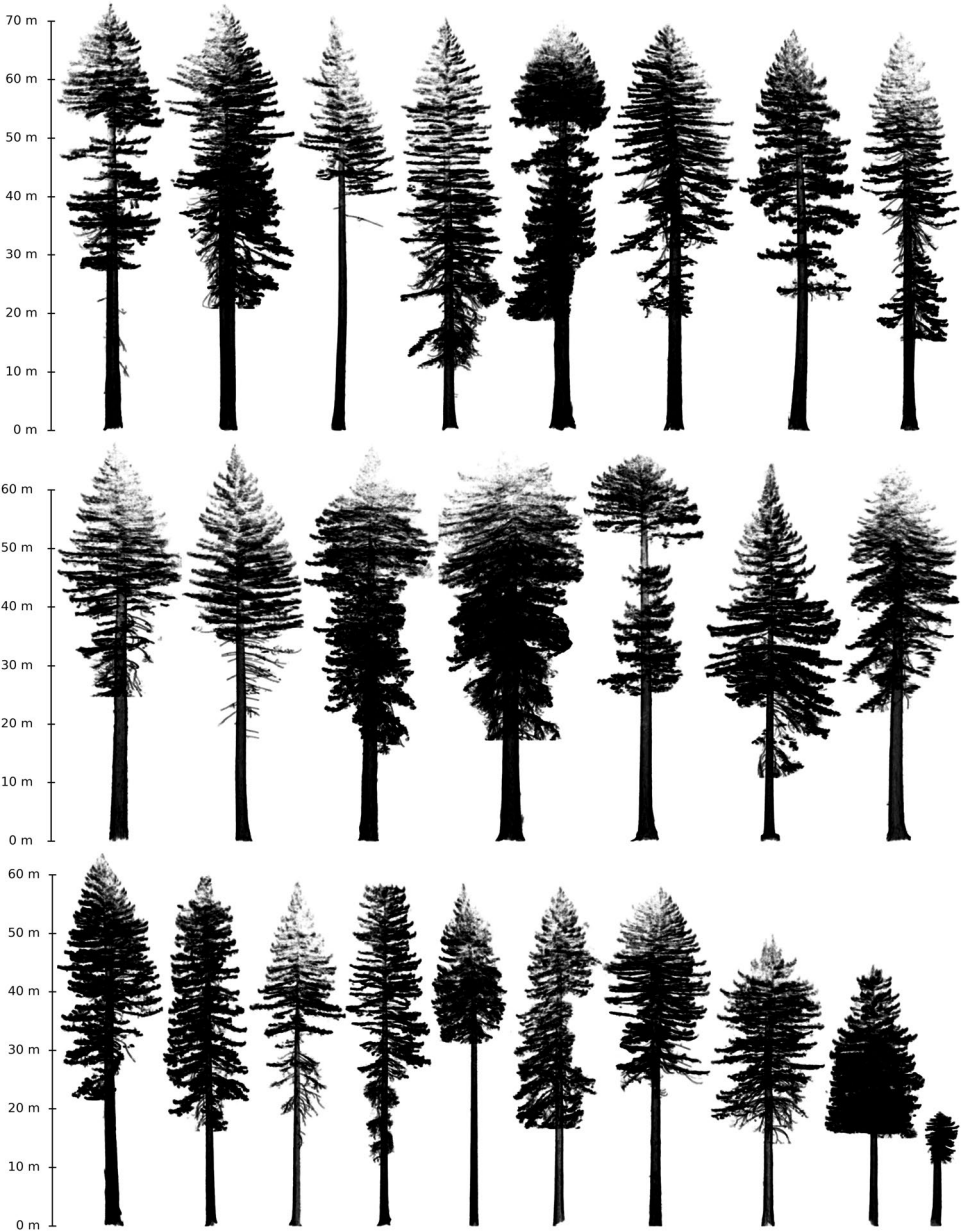
Point clouds of all trees from site CAL-01 (described below).

**Figure 2:**
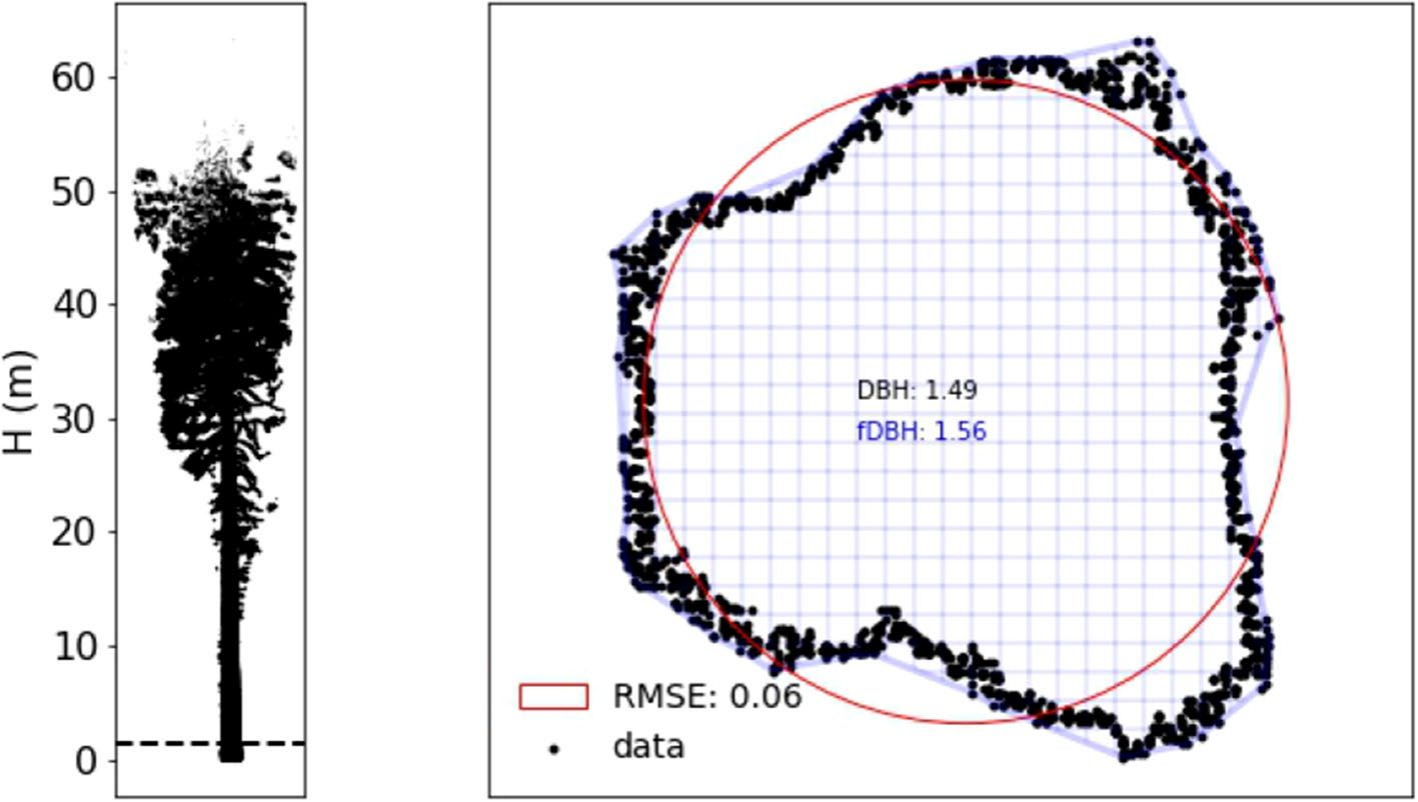
Tree 6 from site CAL-02 (described below): (**left**) side elevation; (**right**) cross section through point cloud at location of dotted line in the (**left**) panel i.e. 1.2–1.4m. The grid shows an alpha shape fit to the TLS cross-section (described below), with the resulting RMSE of fit; and fDBH, the so-called functional DBH, defined as the DBH of a circle of equivalent area to the estimated non-circular trunk cross-sectional area.

**Figure 3:**
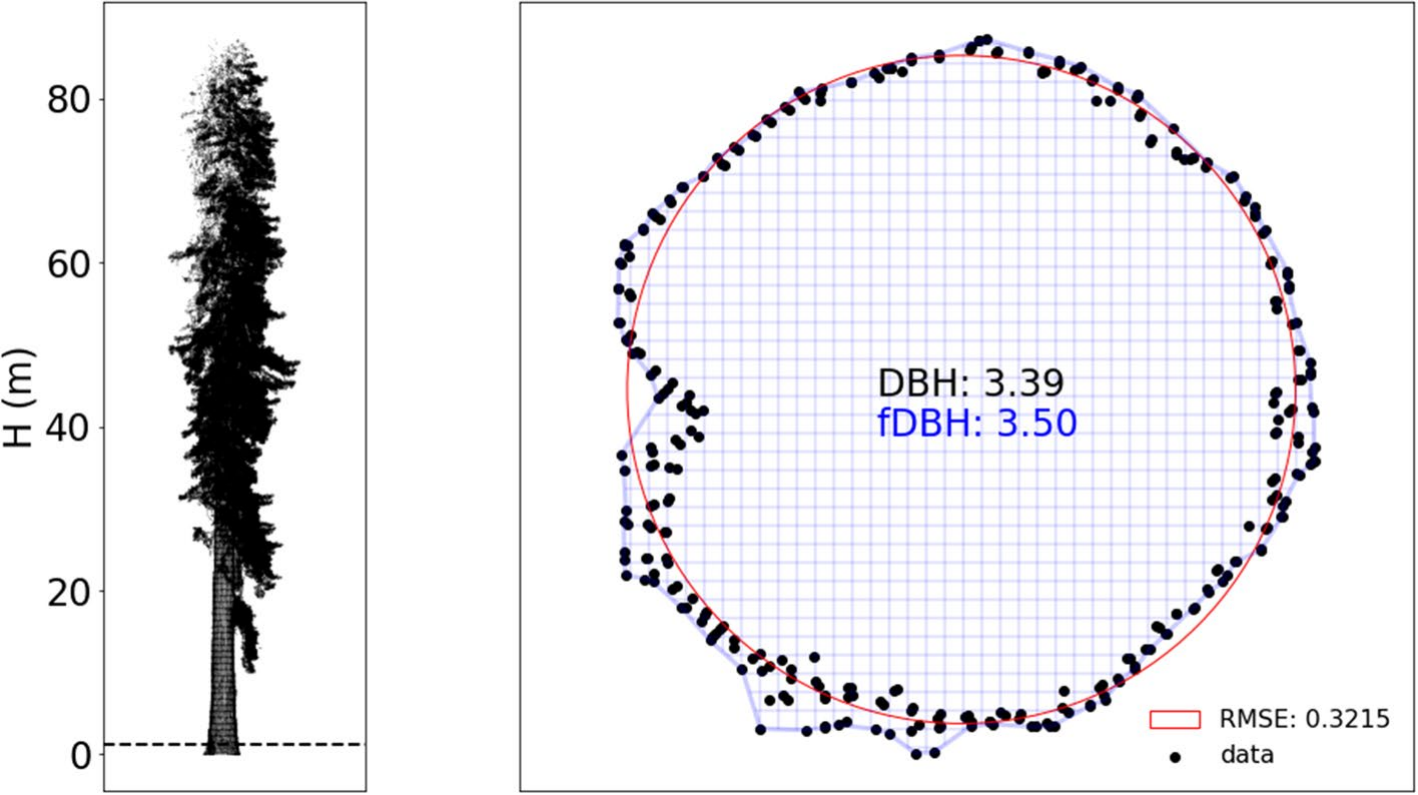
The Colonel Armstrong tree: (**left**) side elevation; (**right**) cross section through point cloud as above.

Point clouds of trees extracted from a single plot (CAL-01, below) are shown in Fig. 1, illustrating the diversity of tree size and form of trees growing within a few m of each other under the same environmental conditions. Cross sections through the point clouds of two trees are shown in Figs. 2 and 3. This illustrates the potential variety of trunk and crown shapes and nature of the bark roughness. Note the different scales in each case.

The Colonel Armstrong tree shown in Fig. 3 is the largest tree we scanned. The TLS point cloud has  14.5 M points, and the tree as measured by TLS is ~88 m tall, with DBH of 3.39 m. The mean and SD of the TLS-estimated volume of the tree is $321\pm 27\,\hbox { m}{^3}$ (see “Methods” below for details). Of this volume,  $216\,\hbox { m}{^3}$ is from the trunk, with the remainder in the branches. The total surface area of the woody part of the tree is $2358\,\hbox { m}{^2}$.

The largest Sequoia by volume anywhere (estimated from trunk diameter) is apparently the General Sherman, with a reported volume of $1487\,\hbox { m}{^3}$. Interestingly, while the Colonel Armstrong would not even get close to the list of the top 30 largest *Sequoia sempervirens* by volume, at only 35% of the 30th placed tree, it is taller than every one of those trees. This is indicative of the variation in H:DBH that these trees can encompass. It also rather raises the question of how well an allometry based on DBH, or DBH and H, is able to predict AGB of large trees.

**Figure 4:**
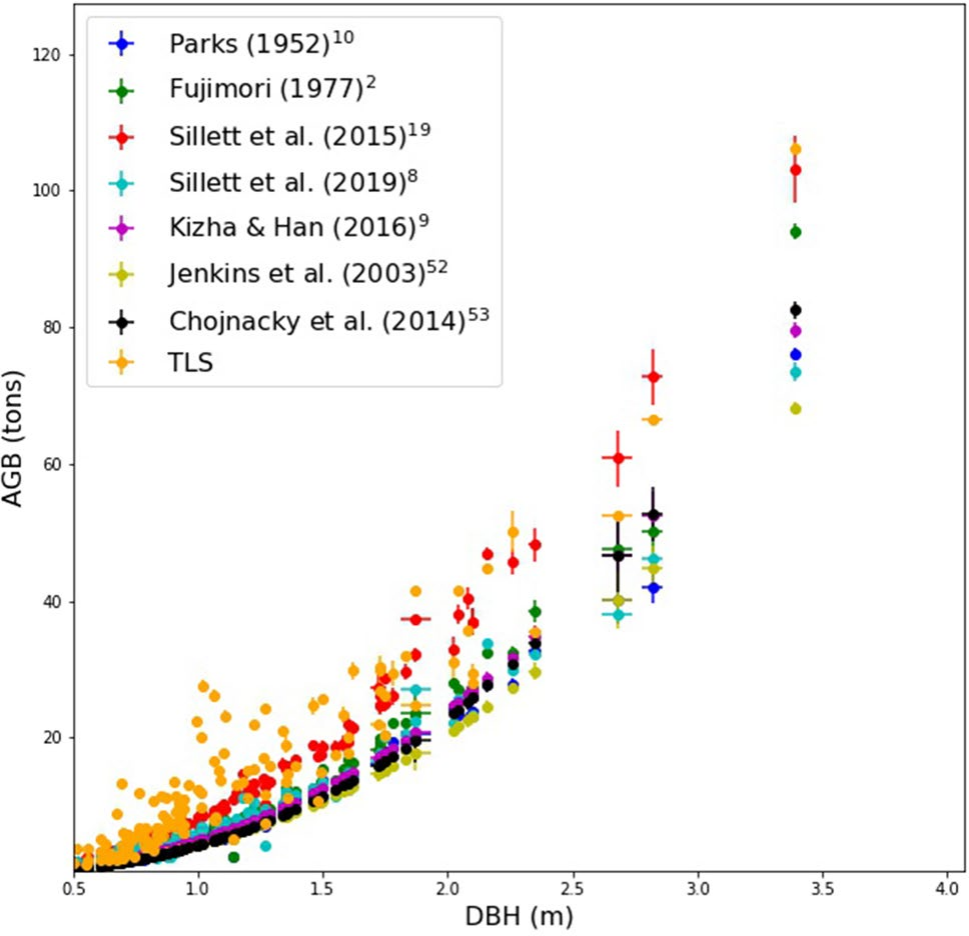
AGB estimated from all allometries, as a function of DBH.

Figure 4 shows the various allometric model estimates of AGB varying with DBH as well as the TLS-derived AGB values. The variation in allometric AGB is much greater (in an absolute sense; the Sillett et al.^19^ estimate is 78% larger than the Jenkins et al.^52^ estimate) for the largest tree than for the others, varying between 70 (Jenkins et al.^52^ and 103 tons (Sillett et al.^19^), against a TLS-derived value of 106 tons. Larger relative differences occur, particularly for trees between 10 and 30 tons—up to nearly 100% in some cases. It is also clear that the TLS and crown-mapping estimates are higher than the species-specific or generalised allometries, the latter being considerably lower.

**Figure 5:**
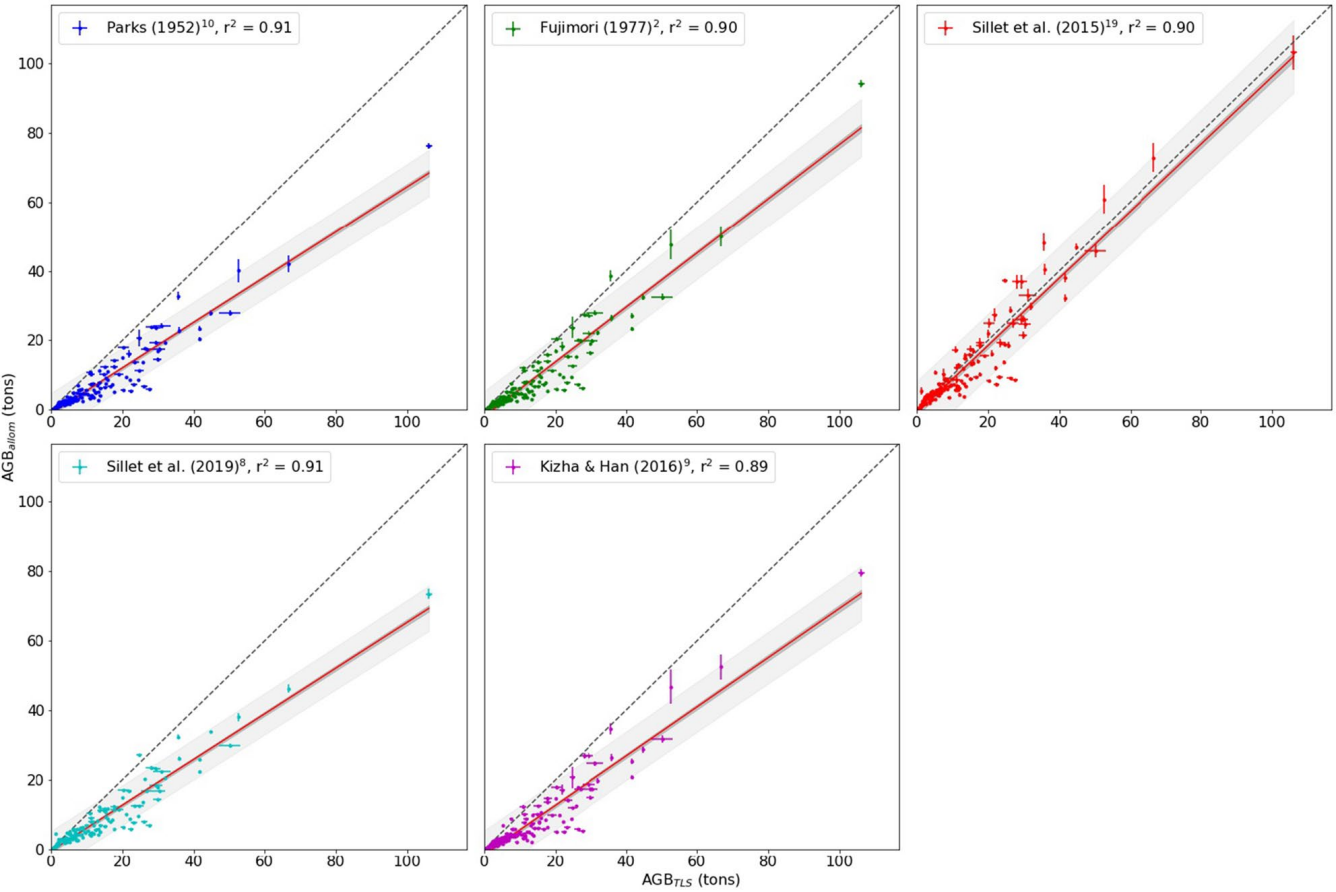
Comparison between TLS-derived and allometric estimates of AGB from species-specific allometries only, using the same wood density. 95% CI and PIs are shown as dark and light gray shading, respectively.

**Figure 6:**
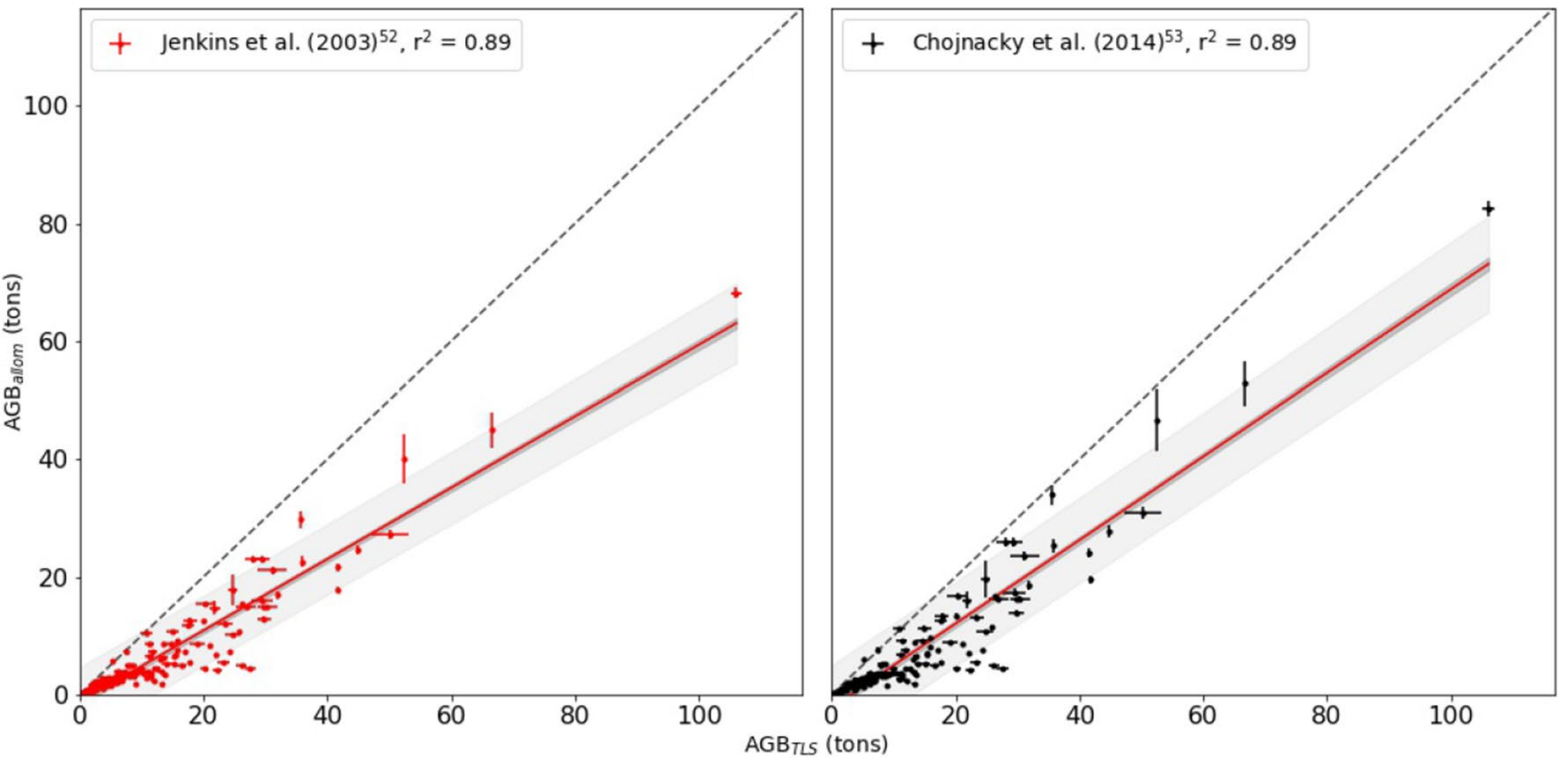
Comparison between TLS-derived and allometric AGB estimated from generalised allometries only. 95% CI and PIs are shown as dark and light gray shading, respectively.

Figure 5 shows the regressions of TLS and species-specific allometric estimates of AGB. Figure 6 shows the comparison between TLS-derived and allometric AGB estimated from generalised allometries only. Table 1 summarises the resulting model fits from all allometric models both including and excluding the Colonel Armstrong tree, to quantify the impact of a single large tree on an allometric regression model. The species-specific allometric and generalised allometry results are grouped separately. Plot-level AGB density (AGBD) values for all plots are given in Table 2, for both TLS-derived and allometric estimates. AGBD is the total AGB for all trees in each plot divided by plot area.

To demonstrate how crown mapping and TLS might be used to estimate additional canopy properties related to AGB and function, we used the crown depth and volume approximation of Sillett et al.^8^ to estimate crown volume (CV), and the number and mass of needles for the Colonel Armstrong tree. The resulting crown volume (CV) is $5378\,\hbox { m}{^3}$, with 168 million needles, needle mass 320 kg, needle area $2100\,\hbox { m}{^2}$. The tree most comparable in size measured by^19^ is their tree SESE21, with $\hbox {H} = 86.10$ m and functional DBH, $\hbox {fDBH} = 349$ cm, weighing 55.59 tons. That tree has $\hbox {CV} = 7755\hbox { m}{^3}$, needle mass = 546.3 kg, needle area of $3068\hbox { m}{^2}$ and 298 million needles.

**Table 1:** Allometric model fits to DBH and fDBH derived from TLS, with the TLS-derived AGB used as reference.

Model	Slope	Intercept	$\mathbf{r} ^{2}$	RMSE (tons)	CV (%)
**With Colonel Armstrong tree**					
Parks^10^	0.65	$-$ 0.99	0.91	35.35	2.63
Fujimori^2^	0.78	$-$ 1.84	0.90	43.62	3.24
Sillett et al.^19^	0.97	$-$ 0.85	0.90	55.04	4.09
Sillett et al.^8^	0.66	$-$ 0.46	0.91	34.42	2.56
Kizha and Han^9^	0.71	$-$ 1.48	0.89	41.63	3.09
Jenkins et al.^52^	0.61	$-$ 1.26	0.89	35.66	2.65
Chojnacky et al.^53^	0.71	$-$ 2.16	0.89	43.08	3.20
**Without Colonel Armstrong tree**					
Parks^10^	0.62	$-$ 0.57	0.87	34.08	2.66
Fujimori^2^	0.72	$-$ 1.16	0.86	40.84	3.19
Sillett et al.^19^	0.96	$-$ 0.79	0.86	55.02	4.30
Sillett et al.^8^	0.64	$-$ 0.23	0.87	34.04	2.66
Kizha and Han^9^	0.68	$-$ 1.16	0.84	41.00	3.20
Jenkins et al.^52^	0.58	$-$ 0.99	0.84	35.13	2.74
Chojnacky et al.^53^	0.67	$-$ 1.65	0.83	41.58	3.25

Fits are across all plots with and without Colonel Armstrong Tree, species-specific allometry first, followed by generalised and including RMSE of model fit and coefficent of variation (CV %).

**Table 2:** AGBD for all plots (t ha$^{-1}$).

Model	CAL-01		CAL-02		CAL-07	
	AGBD	±	AGBD	±	AGBD	±
TLS	2593	115	2252	128	2361	117
Parks^10^	1554	52	1505	45	1090	14
Fujimori^2^	1778	64	1686	54	1135	15
Sillett et al.^19^	2504	111	2301	118	1709	78
Sillett et al.^8^	1680	29	1525	28	1320	17
Kizha and Han^9^	1666	71	1578	58	1578	58
Jenkins et al.^52^	1428	61	1353	50	862	13
Chojnacky et al.^53^	1578	74	1454	58	838	14

Uncertainty is estimated as the standard error of QSM volume fit for the TLS values and the standard error of model fit to TLS-derived DBH, fDBH in the case of the allometric models.

## Discussion

This is (we believe) the first time that TLS has been used to make full volume measurements of large *Sequoia sempervirens* trees. Our results suggest that TLS is well-suited to making detailed 3D structural measurements of the size and volume of these trees. The TLS measurements were collected in a few days per ha and sample the full size range of trees in each plot.

Our TLS-derived estimates of volume and AGB agree closely with an allometric model developed from detailed crown mapping measurements of large *Sequoia sempervirens* trees^19^. In this case TLS-derived and allometric AGB agree to within 2% AGB and, crucially, with no difference in regression slope. All other allometric models tested, based on samples of smaller trees and/or from more indirect measurements, tend to under-estimate AGB compared to both TLS and crown-mapping estimates, with generalised allometries being 30–40% lower. The fit in each of these case is unsurprisingly similar given the form of the allometric models, but the slope and intercept differences indicate calibration data with different properties to those provided by TLS and crown mapping. The fact that TLS and crown mapping agree so closely is encouraging. The estimates of volume are completely independent (AGB estimates share wood density) with their own pros and cons, but that also means their uncertainties will be uncorrelated. It has been suggested that crown mapping has two key advantages over TLS: the ability to estimate leaf components from crown dimensions and branch allometries; and the ease with which it can be applied to giant trees in remote areas^8^. However, TLS measurements also allow retrieval of crown and branch size information^38^. As we show above, there is also the possibility to use TLS with crown mapping to estimate crown volume, needle mass and number. By definition, these numbers are impossible to validate—much more so than the woody volume and mass—based as they are on very large extrapolation, and should be considered as indicative at best. However, if validated these properties are potentially useful in developing allometries based on crown volume, accounting for foliar biomass in AGB, and relating to photosynthesis and transpiration. In terms of the difficulty of collecting TLS data, it has already been used to measure the structure of very large trees (H up to 100 m) in remote tropical forest^34,35,39^.

Verbeeck et al.^40^show that large trees may have more variable architecture than smaller ones on a so-called structural economics spectrum (SES), due to greater variations of crown morphology. Routinely combining the structural information provided by TLS with the type of branch-level allometry developed via crown mapping for leaf material^8^, would potentially provide the best of both worlds in terms of understanding relationships between architecture and function in these very large trees.

Our results suggest that TLS is well-suited to characterising the size and mass of *Sequoia sempervirens* trees, particularly if other measurements are hard to make. This is of particular importance when attempting to calibrate and validate estimates of AGB which are made from EO^30,41^. Our results also suggest that allometries developed from less detailed or non species-specific measurements, are likely to significantly underestimate biomass in these tall forests with exceptionally high AGBD. The TLS-derived estimates of AGBD include all trees in a plot, whereas allometric estimates are based on selected individuals are not spatially explicit. This is of particular importance to upscaling from tree-based to area-based estimates of AGB, for both EO and census-based estimates. While uncertainty in the individual tree AGB estimates will be reduced at these aggregate scales, bias in the estimates would not. If allometric models yield biased estimates of plot-level AGB, these biases will naturally be passed to the satellite models. Our results suggest that the TLS individual tree estimates do not have biases with respect to species-specific allometric models, but show systematic bias with respect to the generalized allometries that are used to train satellite products (e.g. Jenkins et al.^52^).

As discussed above, larger trees have a wider range of AGB, but are rarely measured in allometric samples due to the difficulty of measurement. Samples of much smaller harvested trees will not represent this greater variance with size. In addition, partial measurement of felled or fallen trees will tend to underestimate branch mass. Using measurements of other, smaller species to infer the mass of *Sequoia sempervirens* is also a potential source of underestimate, given they are seemingly exceptionally massive for given DBH.

Table 1 demonstrates the difference a single large tree can have on allometric predictions. The regression $\hbox {r}^2$ values are all higher with the Colonel Armstrong tree included and the slopes are all closer to one, increasing by up to 7% in the case of Fujimori^2^ for example. This shows how a few particularly large trees may dominate an allometric regression model. Or conversely, how failing to sample the full range of tree size risks a biased regression model. Most of the trees we measured here have DBH greater than the largest tree in the sample of Kizha and Han^9^ (max DBH 85 cm). A key advantage of the TLS approach is that it removes the need to select trees *a priori* a i.e. all trees within a given plot are captured. So trees of $< 85$ cm DBH are not representative of the size distribution in the plots measured here. This is perhaps not surprising given that Kizha and Han^9^ measured in commercial forestry plots. But an allometry calibrated using these measurements and then applied to trees in non-commercial forest plots will be extrapolating well outside the DBH:AGB calibration range. This is fine as long as the fundamental allometric assumption of a scale-invariant size-mass relationship holds; if not, this will fail to capture AGB variance of larger trees.

Care needs to be taken in considering the effects of sampling on allometric model selection and uncertainty^17,42^. Even if observations from a particular size class, species or type are present in allometric calibration data, the resulting model is not necessarily suitable for predictions even within those groups if the calibration data are overwhelmed by observations from other types (e.g. far more trees of one size class than another)^17^.

And whilst it is true that out-of-sample predictions of trees outside the range of the in-sample (calibration) data may in general be ‘a bad thing’, particularly if the assumptions of allometric regression are violated, this raises an interesting question: how do we resolve the seeming contradiction between these warnings on the one hand, and the fundamental assumption of scale-invariance that underpins the concept of allometry on the other i.e. that big trees have the same diameter-mass relationship as smaller ones^43,44^? That aside, the disproportionate impact of large trees also indicates that more estimates of the volume of large trees are needed, at least commensurate with their contribution to AGB if that is the property of interest. Whether these are obtained by TLS, crown mapping or other methods, they will help avoid errors caused by undersampling and allow better characterisation of variance in allometric predictions of AGB at larger DBH. This is of particular importance for EO estimates of AGBD, given the relatively weaker relationship between H and AGB than between DBH and AGB^45^.

## Methods

### Study sites

TLS data were collected during a campaign in 3 locations in Sonoma County, California during 2017 at three sites, listed below.

- Grove of Old Trees, CAL-01: 38.4, − 123.0;
- Armstrong Redwoods State Natural Reserve, CAL-02: 38.5, − 123.0;
- Sea Ranch, CAL-07: 38.7, − 123.5

See https://mattbv.github.io/tls-data-map/ for more location details. The climate in Sonoma County is broadly temperate coastal, with annual average rainfall of 80 cm, annual average temperature of $15 \,^\circ \hbox {C}$, with mean low of $7 \,^\circ \hbox {C}$ and mean high of $23\, ^\circ \hbox {C}$. Prevailing westerly winds bring a large amount of moisture inland as sea mist, which is crucial to the existence of *Sequoia sempervirens*^9^.

### TLS data collection and processing

TLS data were collected with a Riegl VZ-400 TLS (RIEGL Laser Measurement Systems GmbH, Austria) using $0.04^\circ$ scan resolution, and upright and $90^\circ$ tilt scans at each location, with the scanner mounted at 1.5 m on a tripod. The scanner range is ~700 m, with beam divergence 0.35 mrad leading to a footprint diameter of 42 mm at 100 m. Scans were taken on a 12.5 m grid using multiple reflectance targets to enable co-registration of scans, following the protocol of^46^. For the Colonel Armstrong tree, scans were collected at five locations around the tree at a distance of 10–20 m. All TLS scans were then co-registered into plot-level point clouds using the Riegl RiSCAN PRO software package (v 2.7.1). Clusters of tree points were extracted from each plot point cloud for further analysis using the *treeseg* tool^47^. All tree measurements referred to here are *Sequoia sempervirens* only.

#### Grove of Old Trees, CAL-01

The Grove of Old Trees is a 13.5 ha region of old-growth redwoods, privately-owned and managed by non-profit local organisations. The site is in the so-called Petaluma Wind Gap that carries cooler, wetter air from the coast inland and provides the moist climate required for large *Sequoia sempervirens* growth. A 1 ha area was scanned within the larger site and of this the central 0.25 ha was used, with twenty six trees extracted from the resulting point cloud. The tree dimensions derived from the TLS point clouds are given in Supplementary Table S1.

#### Armstrong Redwoods State Natural Reserve, CAL-02

This plot was located within Armstrong State Natural Reserve near Guerneville, and was one of five 0.25 ha plots that were positioned to avoid roads, pedestrian paths, enclosed areas and river beds. Thirty six trees were extracted from the resulting point cloud. The Colonel Armstrong tree was just outside the plot and was scanned separately.

#### The Sea Ranch, CAL-07

The Sea Ranch is a privately-owned reserve along 16 km of the Sonoma County coast (https://www.tsra.org). A 1 ha plot was scanned and the central 0.25 ha was extracted for further analysis. Eighty two trees were extracted from the resulting point cloud. This plot contained characteristic ‘fairy ring’ structures of secondary growth stems growing around the site of an older, absent tree. Fairy rings have generally been thought to arise mainly (but not exclusively) from clonal spread, a key (and unusual in conifers) reproductive strategy for *Sequoia sempervirens*^48^.

### TLS and allometric estimates of volume and AGB

The volume of each tree was estimated by fitting Quantitative Structural Models (QSM) using the TreeQSM approach of^49^. This provides an estimate of tree volume (as well as branch and trunk size distributions) of each extracted tree point cloud. For each tree, multiple QSMs were fitted to each point cloud, using the parameter optimisation approach of^47^, resulting in a mean and standard deviation of QSM volume estimation. To estimate DBH from the TLS point clouds, a cross-section was taken through each point cloud at 1.2–1.4m above the lowest point (indicated by the dotted lines in Figs. 2 and 3). Each 20 cm thick slice was collapsed onto a plane and a least squares circle-fitting algorithm applied^50^. The RMSE of the circle fit was used in the subsequent allometric equations to estimate the uncertainty in AGB due to DBH estimation.

We convert TLS-derived estimates of volume (via QSM fitting) to AGB using the same wood density values as those in five allometric models developed specifically for *Sequoia sempervirens*, and two general (non species-specific) models. We then compare our TLS-derived estimates of tree AGB with these allometric model estimates of AGB. To generate the latter, we fit the published forms of these models (i.e. using the specified model parameter values), to size and shape variables derived from TLS i.e. DBH and variants thereof, as well as tree height, H. H here is taken from the highest point of the original point cloud in each case. The explicit form of each model, and the parameter values used in each case, are given in Supplementary Table S1.

#### Species-specific models

Fujimori^2^ and Parks^10^ developed species-specific DBH, H allometries based on volume estimates of larger trunk and branch components sampled from fallen trees i.e. non-destructive, partial samples. We also compare with two more recent allometric models developed by Sillett et al.^8,19^, which predict AGB and total volume. The 2015 model was developed from detailed 3D crown mapping via tree-climbing measurements. These were used to generate estimates of 3D volume of as many of the components of large *Sequoia sempervirens* trees as possible, and also included multiple cores for wood density and specific gravity. Empirical relationships between sampled branch volume and leaf mass were also generated. The later model used the previous crown mapping structural data to develop an allometry of the same form, but using ground-based measurements of DBH, H and crown size.

For the^19^ model of predicted AGB, the independent variables are diameter at the top of buttress (DTB) and functional DBH (fDBH). Total wood volume is predicted using the same form but with different coefficients. The TLS-derived values of DTB, fDBH are given in Supplementary Tables S2–S4. Across all trees, the difference between DBH and fDBH varies by mean − 6.9%, stdev 2.6% (i.e. fDBH > DBH). Figure 2 shows that the difference between DBH and fDBH may only be a few % by chance despite potentially large departures from the assumption of a circular cross-section. For the^8^ model, the independent variables are DTB and H.

To estimate DTB, Sillett et al.^19^ plotted their measurements of tree circumference (x, y) and then estimated the resulting cross-sectional area via an image-processing approach. Here, we estimate DTB by taking a circle fitted to points in the range 4–4.5 m up the trunk to guarantee being above buttresses. We estimate fDBH by fitting a concave hull (alpha shape) (Python alphashape toolbox v 1.0.1^51^). We used an alpha parameter of 4.0 determined as a balance between fitting more (higher alpha) or less (lower alpha) tightly, to the cloud sections. The resulting alpha shape areas are shown in blue hatching in Figs. 2 and 3, and the circle of equivalent area is then used to estimate fDBH. The RMSE of fit is used as the uncertainty in the resulting fDBH and propagated through the allometric volume estimates. We also fitted the^8^ model to the TLS-derived estimates of volume, to generate a new set of model parameters based on the 146 extracted TLS trees. The resulting RMSE of model fit is 1.33% (compared to 1.44% when using the original^8^ parameters). As an example of how these measurements might be used for EO estimates of volume and AGB, an H-only allometry derived from TLS predicts volume with RMSE of ~20% (see Supplementary Fig. S1).

The other species-specific allometry we compare with is that of Kizha and Han^9^, the only one developed using destructive harvest and weighing. They worked in commercial logging sites, sampling smaller trees in the DBH range 2.54–84 cm. Trunk volume was estimated by section diameters up the trunk and mass was sampled via disks. Branch mass was sampled for various size classes, as well as foliage and live/dead fractions and empirical relationships with size developed for each. In this way the total wood volume and mass were estimated from each tree. The resulting model is the only species-specific allometry here that uses DBH only (along with a correction factor for bias in back-transformation to arithmetic units).

#### Generalised allometric models

We also compare TLS with two generalised (i.e. non-species specific) allometric models. These are the models of Jenkins et al.^52^ and the closely-related model of Chojnacky et al.^53^, which have been widely-used in the US to estimate regional AGB. Both models take the same form, and the model parameters are determined by species class. For Jenkins et al.^52^ *Sequoia sempervirens* is classified in the cedar/larch group. For Chojnacky et al.^53^ *Sequoia sempervirens* is classified in the *Cupressaceae* 0.30–0.39 spec. grav. group.

The Jenkins et al.^52^ model was developed from meta-analysis of more than 2000 published DBH-based allometric models for U.S. trees, in order to develop a set of national-scale AGB regression equations. This approach groups species into softwood and hardwood categories based on a combination of taxonomic relationships, wood specific gravity, and DBH-to-AGB relationships. An issue with this model is that in order to overcome the relatively small sample range of DBH, a form of bootstrapping was used to generate ‘pseudo data’ from the original samples. The resulting allometric model was then fitted to these pseudo data, which implies a fit to an existing fitted model. It is not clear how the resulting uncertainty is dealt with in this case.

The Chojnacky et al.^53^ model is based on the same underlying data as Jenkins et al.^52^ but re-analysed at DBH intervals within the DBH ranges of the original equations, and considering the similarity (or not in some cases) of the specific gravity of tree species within genera and/or families.

## Supplementary information

Supplementary Information

## References

[1] Van Pelt R, Sillett SC, Kruse WA, Freund JA, Kramer RD (2016). Emergent crowns and light-use complementarity lead to global maximum biomass and leaf area in Sequoia sempervirens forests. For. Ecol.Manag..

[2] Fujimori T (1977). Stem biomass and structure of a mature sequoia sempervirens stand on the Pacific Coast of Northern California. J. Jpn. For. Soc..

[3] Busing RT, Fujimori T (2005). Biomass, production and woody detritus in an old coast redwood (Sequoia sempervirens) forest. Plant Ecol..

[4] Koch GW, Sillett SC, Jennings GM, Davis SD (2004). The limits to tree height. Nature.

[5] Carder AC (1995). Forest Giants of the World, Past and Present.

[6] Harrison JG, Forister ML, Parchman TL, Koch GW (2016). Vertical stratification of the foliar fungal community in the world’s tallest trees. Am. J. Bot..

[7] Sillett SC (2010). Increasing wood production through old age in tall trees. For. Ecol. Manag..

[8] Sillett SC (2019). Allometric equations for Sequoia sempervirens in forests of different ages. For. Ecol. Manag..

[9] Kizha AR, Han H-S (2016). Predicting aboveground biomass in second growth coast redwood: Comparing localized with generic allometric models. Forests.

[10] Parks, W.H. Redwood log characteristics: Sapwood thickness, bark thickness and log taper. Report number 1.20121. California Redwood Association, San Francisco (1952).

[11] Keith H, Mackey BG, Lindenmayer DB (2009). Re-evaluation of forest biomass carbon stocks and lessons from the world’s most carbon-dense forests. Proc. Nat. Acad. Sci..

[12] Slik JF (2013). Large trees drive forest aboveground biomass variation in moist lowland forests across the tropics. Glob. Ecol. Biogeog..

[13] Lindenmayer DB, Laurance WF (2016). The ecology, distribution, conservation and management of large old trees. Biol. Rev..

[14] Rüger N (2020). Demographic trade-offs predict tropical forest dynamics. Science.

[15] Chave J (2005). Tree allometry and improved estimation of carbon stocks and balance in tropical forests. Oecologia.

[16] Chave J (2014). Improved allometric models to estimate the aboveground biomass of tropical trees. Glob. Change Biol..

[17] Burt A (2020). Assessment of bias in pan-tropical biomass predictions. Front. For. Glob. Change Trop. For..

[18] Sillett SC, Van Pelt R, Kramer RD, Caroll AL, Koch GW (2015). Biomass and growth potential of *Eucalyptus regnans* up to 100 m tall. For. Ecol. Manag..

[19] Sillett SC (2015). How do tree structure and old age affect growth potential of California redwoods?. Ecol. Monog..

[20] Kramer RD, Sillett SC, Van Pelt R (2018). Quantifying aboveground components of *Picea sitchensis* for allometric comparisons among tall conifers in North American rainforests. For. Ecol. Manag..

[21] Niklas KJ (1993). Influence of tissue density-specific mechanical properties on the scaling of plant height. Ann. Bot..

[22] Chave J (2009). Towards a worldwide wood economics spectrum. Ecol. Lett..

[23] Luxford RF, Markwardt LJ (1932). The strength and related properties of redwood. USDA Tech. Bull..

[24] Wilson PL, Funck WJ, Avery RB (1987). Fuelwood characteristics of northwestern conifers and hardwoods. Res. Bull..

[25] Miles, P. D. & Smith, B. Specific gravity and other properties of wood and bark for 156 tree species found in North America. Res. Note NRS-38. Newtown Square, PA: U.S. (2009), Department of Agriculture, Forest Service, Northern Research Station, p. 35.

[26] Clark DB, Kellner JR (2012). Tropical forest biomass estimation and the fallacy of misplaced concreteness. J. Veg. Sci..

[27] Momo ST (2018). Using volume-weighted average wood specific gravity of trees reduces bias in aboveground biomass predictions from forest volume data. For. Ecol. Manag..

[28] Réjou-Méchain M, Tanguy A, Piponiot C, Chave J, Hérault B (2017). Biomass: An r package for estimating above-ground biomass and its uncertainty in tropical forests. Methods Ecol. Evol..

[29] Chave, L. *et al.* Ground data are vital for remote sensing missions. In *Surveys in Geophysics vol 71: Forest Biomass and Structure from Space* (eds Scipal, K., Dubyah, R., Le Toan, T., Quegan, S., Cazenave, A., Lopez, T.) 40 (4), 863–880 (2019).

[30] Duncanson, L. *et al*. The importance of global land product validation: Towards a standardized protocol for aboveground biomass. In *Surveys in Geophysics vol 71: Forest Biomass and Structure from Space*. (eds Scipal, K., Dubyah, R., Le Toan, T., Quegan, S., Cazenave, A., Lopez, T.) **40 (4)**, 979–999 (2019). 10.1007/s10712-019-09538-8 PMC6647371 31395994

[31] Disney MI (2018). Terrestrial LiDAR: A 3D revolution in how we look at trees. New Phytol..

[32] Disney MI (2018). Weighing trees with lasers: Advances, challenges and opportunities. R. Soc. Interface Focus.

[33] Calders K (2015). Nondestructive estimates of above-ground biomass using terrestrial laser scanning. Methods Ecol. Evol..

[34] Gonzalez de Tanago J (2018). Estimation of above-ground biomass of large tropical trees with terrestrial LiDAR. Methods Ecol. Evol..

[35] Momo Takoudjou S (2018). Using terrestrial laser scanning data to estimate large tropical trees biomass and calibrate allometric models: A comparison with traditional destructive approach. Methods Ecol. Evol..

[36] Stovall AE, Anderson-Teixeira KJ, Shugart HH (2018). Assessing terrestrial laser scanning for developing non-destructive biomass allometry. Forest Ecol. Manag..

[37] Disney, M. I., Burt, A., Calders, K., Schaaf, C. & Stovall, A. Innovations in ground and airborne technologies as reference and for training and validation: Terrestrial laser scanning (TLS). In *Surveys in Geophysics vol 71: Forest Biomass and Structure from Space*. (eds Scipal, K., Dubyah, R., Le Toan, T., Quegan, S., Cazenave, A., Lopez, T.) **40 (4)**, 937–958 (2019).

[38] Lau A (2018). Quantifying branch architecture of tropical trees using terrestrial LiDAR and 3D modelling. Trees.

[39] Shenkin A (2019). The world’s tallest tropical tree in three dimensions. Front. For. Glob. Change..

[40] Verbeeck H (2019). Time for a plant structural economics spectrum. Front. For. Glob. Change..

[41] Duncanson L, Huang W, Johnson K, Swatantran A, McRoberts RE, Dubayah R (2017). Implications of allometric model selection for county-level biomass mapping. Carbon Balance Manag..

[42] Sileshi GW (2014). A critical review of forest biomass estimation models, common mistakes and corrective measures. For. Ecol. Manag..

[43] Enquist BJ (2002). Universal scaling in tree and vascular plant allometry: Toward a general quantitative theory linking plant form and function from cells to ecosystems. Tree Physiol..

[44] Niklas KJ (2006). A phyletic perspective on the allometry of plant biomass-partitioning patterns and functionally equivalent organ-categories. New Phytol..

[45] Hunter MO, Keller M, Victoria D, Morton DC (2013). Tree height and tropical forest biomass estimation. Biogeosciences.

[46] Wilkes P (2017). Data acquisition considerations for terrestrial laser scanning of forest plots. Rem. Sens. Environ..

[47] Burt A, Disney MI, Calders K (2018). Extracting individual trees from lidar point clouds using treeseg. Methods Ecol. Evol..

[48] Douhovnikoff, V. & Dodd, R. S. Clonal spread in second growth stands of coast redwood, sequoia sempervirens. In: Standiford, R. B. et al., technical editors. *Proceedings of the Redwood Region Forest Science Symposium 2007: What Does the Future Hold? Gen. Tech. Rep. PSW-GTR-194. Albany, CA: Pacific Southwest Research Station, Forest Service, US Department of Agriculture. * Vol 194, 65–72 (2007).

[49] Raumonen P (2013). Comprehensive quantitative tree models from terrestrial laser scanner data. Remote Sens..

[50] Olofsson K, Holmgren J, Olsson H (2014). Tree stem and height measurements using terrestrial laser scanning and the RANSAC algorithm. Remote Sens..

[51] Bellock, K. E. Alphashape Python toolbox, v 1.0.1. https://pypi.org/project/alphashape/ (2019).

[52] Jenkins JC, Chojnacky DC, Heath LS, Birdsey RA (2003). National-scale biomass estimators for United States tree species. For. Sci..

[53] Chojnacky DC, Heath LS, Jenkins JC (2014). Updated generalized biomass equations for North American tree species. Forestry.

