# Supplementary material for: New 3D measurements of large redwood trees for biomass and structure
Source: Sci Rep. 2020 Oct 15;10:16721. doi: 10.1038/s41598-020-73733-6 (PMC7566452; doi:10.1038/s41598-020-73733-6)
Supplement: Supplementary file 1 — Supplementary Information [file 41598_2020_73733_MOESM1_ESM.pdf]

Supplementary: New 3D measurements of large redwood trees for biomass and structure

September 1, 2020

Mathias Disney, John Armston, Andrew Burt, Laura Duncason and Phil Wilkes
m.disney@ucl.ac.uk

Allometric model details

Table S1 shows the details of all published and TLS-derived (this study) models used. For the previously published models all coefficients a-d are taken direct from the publications. For the TLS-derived DTB, H model, model coefficients are obtained by fitting the Sillett et al. (2019)[8] model to the TLS-derived estimates of volume using TLS estimates of DTB and H. The same is true for the H only model with the given form.

Table S1: Allometric model forms and parameter values. See Methods for definitions of DTB and fDBH.

**Model**	**Form**	**a**	**b**	**c**	**d**
Parks (1952)[10]	$log_{10}(Vol) = alog_{10}(DBH^2H) - b$	0.9246	0.4147		
Fujimori (1977)[2]	$log_{10}(Vol) = alog_{10}(DBH^2H) - b$	0.9784	0.4843		
Sillett et al. (2015)[19]	$AGB = aDTB^b + cfDBH^d$	3.9656×10^{-4}	2.3122	1.9583×10^{-3}	1.8657
Sillett et al. (2015)[19]	$Vol = aDTB^b + cfDBH^d$	2.2529×10^{-4}	2.3747	1.2574×10^{-3}	1.9171
Sillett et al. (2019)[8]	$AGB = aDTB^b + cH^d$	7.585×10^{-2}	2.401	3.468×10^{-3}	3.301
Sillett et al. (2019)[8]	$Vol = aDTB^b + cH^d$	2.752×10^{-4}	2.372	1.173×10^{-5}	3.306
TLS-derived	$Vol = aDTB^b + cH^d$	7.667×10^{-5}	2.624	0.159	1.040
TLS H only	$Vol = aH^b + c$	1.203×10^{-8}	5.36	4.97	
Kizha & Han (2015)[9]	$log_{10}(AGB) = a + blog_{10}(DBH) + c$	-0.8252	2.2607	0.0054	
Jenkins et al. (2003)[51]	$ln(AGB) = a + bln(DBH)$	-2.0336	2.2592		
Chojnacky et al. (2014)[52]	$ln(AGB) = a + bln(DBH)$	-2.7765	2.4195		

Size of trees in all plots

Figure S1 (left panel) shows the fit of VOL:H allometry derived from the TLH estimates of H, with the model parameters in Table S1. The right panel shows the scatter plot of volume estimated using TLS-derived H-only allometry, with the full Sillett et al. (2015)[19] allometry. This is included as a demonstration of the ability of a H-only allometry based on the TLS data to predict the volume of *Sequoia sempervirens* trees. This relationship would potentially allow Earth Observation-derived estimates of H to be used to estimate volume (and AGB). Unsurprisingly, the model fit is worse than that of the DBH, H models (with large uncertainty in the 60-70m height range), but with greater model parsimony.

Tables S2-S4 show the size of all *Sequoia sempervirens* trees extracted from plots at each of the three sites, in height order.

Table S2: Size of trees in Grove of Old Trees, plot CAL-01.

**x (m)**	**y (m)**	**H (m)**	**DBH (m)**	**DTB (m)**	**fDBH (m)**
4.89	5.86	19.57	1.14	1.02	1.23
15.75	17.16	44.71	1.10	1.00	1.18
17.74	19.55	50.04	1.26	1.18	1.33
14.32	18.62	57.99	1.34	1.23	1.49
14.25	15.69	58.01	1.20	1.16	1.27

13.22	13.59	58.57	1.15	1.09	1.24
14.66	12.84	58.60	1.29	1.13	1.36
16.47	14.97	58.85	1.01	0.98	0.48
13.55	14.86	59.94	1.27	1.17	1.33
17.17	18.10	63.71	2.08	1.92	2.22
11.30	16.16	64.29	1.39	1.26	1.50
19.09	15.40	64.59	1.22	1.06	1.30
16.57	13.09	66.15	1.73	1.57	1.81
20.00	20.00	66.65	2.82	2.48	3.04
18.51	17.93	67.68	2.04	1.88	2.14
13.63	16.89	67.83	1.22	1.15	1.30
13.81	17.19	68.10	1.75	1.66	1.85
13.80	14.87	68.11	1.60	1.41	1.73
18.18	14.77	68.69	1.83	1.67	1.91
18.40	19.39	69.58	1.87	1.74	1.97
15.49	15.82	70.17	2.68	2.25	2.87
14.53	16.29	70.66	1.50	1.31	1.60
14.49	15.43	70.94	1.35	1.22	1.45
19.56	17.52	72.90	2.16	2.11	2.26
15.77	16.13	73.34	2.35	2.06	2.48
15.98	15.51	87.79	3.39	2.95	3.49

Table S3: Size of trees in Armstrong State Natural Reserve, plot CAL-02.

x (m)	y (m)	H (m)	DBH (m)	DTB (m)	fDBH (m)
6.60	6.58	15.34	0.24	0.17	0.27
6.37	5.78	19.55	0.25	0.19	0.28
11.10	13.07	36.78	0.78	0.69	0.86
11.99	9.61	36.85	0.88	0.69	0.99
11.40	8.74	37.65	0.83	0.66	0.88
14.28	8.23	38.17	0.88	0.71	0.97
14.81	11.19	38.52	0.89	0.68	0.98
7.03	13.62	41.06	0.91	0.77	0.99
9.71	11.88	47.87	1.27	0.81	1.45
8.37	6.47	51.54	1.27	1.12	1.31
12.96	7.36	53.86	1.06	0.92	1.11
12.30	9.04	53.98	1.07	0.93	1.12
4.95	5.14	55.62	1.20	1.09	1.26
9.08	8.79	55.85	0.77	0.72	0.81
9.09	6.76	55.86	0.93	0.96	0.96
4.25	4.43	56.23	1.48	1.26	1.55
13.67	11.05	59.19	0.73	0.71	0.77
10.14	10.78	59.56	1.55	1.30	1.61
10.40	7.09	60.22	0.79	0.70	0.83
12.37	15.13	60.76	1.46	1.35	1.55
11.79	15.29	61.26	1.18	1.26	1.25
11.93	10.69	62.14	1.36	1.25	1.41
5.32	5.96	63.29	1.36	1.10	0.70
14.93	12.42	63.30	1.49	1.27	1.56
11.29	17.63	63.69	1.72	1.54	1.98
8.35	10.93	63.78	1.58	1.32	1.64
8.63	7.49	63.90	1.60	1.31	1.67
19.33	10.82	64.33	2.10	1.81	2.20
13.15	17.15	64.71	1.62	1.41	1.69
14.04	26.11	64.95	2.10	1.81	2.20
15.36	11.46	66.65	1.06	0.96	1.12
15.55	17.55	66.71	2.26	2.03	2.37
12.41	12.84	68.87	1.73	1.50	1.83

16.24	20.22	68.87	1.73	1.50	1.82
7.35	6.45	69.04	1.75	1.51	1.83
14.37	18.49	70.40	1.87	1.91	2.00
14.29	16.57	71.65	2.02	1.72	2.08
16.06	23.52	72.49	1.78	1.54	1.86

Table S4: Size of trees in Richardson Reserve, plot CAL-07.

**x (m)**	**y (m)**	**H (m)**	**DBH (m)**	**DTB (m)**	**fDBH (m)**
2.20	2.77	19.45	0.27	0.27	0.30
3.28	3.29	23.79	0.35	0.95	0.39
3.40	4.52	28.16	0.55	0.49	0.60
4.12	3.95	32.06	0.44	0.37	0.47
3.25	3.97	34.50	0.43	0.36	0.47
3.69	3.37	34.51	0.39	0.36	0.44
1.52	3.81	37.99	0.74	0.66	0.79
4.60	4.28	38.43	0.47	0.43	0.50
2.63	3.18	40.30	0.26	0.23	0.29
3.02	6.26	41.49	0.56	0.50	0.60
3.71	4.65	41.73	0.47	0.43	0.54
4.74	4.88	41.89	0.82	0.65	0.80
5.12	5.99	42.53	0.56	0.53	0.62
5.99	5.66	42.53	0.61	0.57	0.67
5.01	5.00	43.16	0.63	0.56	0.68
6.24	6.26	43.73	0.65	0.60	0.71
7.35	2.78	43.81	0.50	0.43	0.53
6.72	6.37	43.88	0.69	0.62	0.74
7.69	3.24	45.37	0.70	0.64	0.74
4.84	3.09	45.46	0.48	0.42	0.51
7.69	4.19	45.54	0.64	0.55	0.71
8.15	4.82	46.11	0.79	0.75	0.85
5.66	2.85	46.13	0.68	0.62	0.73
4.36	4.10	46.85	0.72	0.67	0.76
5.97	7.60	46.85	0.84	0.74	0.90
6.39	6.28	46.86	0.72	0.67	0.77
5.42	5.46	46.91	0.79	0.71	0.84
5.57	4.17	46.98	0.70	0.65	0.75
4.93	8.20	47.11	0.79	0.73	0.86
10.12	10.81	47.19	0.86	0.74	0.92
4.19	4.18	47.41	0.68	0.54	0.74
7.44	7.45	47.77	0.71	0.69	0.78
7.59	6.88	47.82	0.76	0.72	0.83
5.65	4.80	47.87	0.61	0.56	0.66
3.59	3.62	48.13	0.70	0.60	0.78
7.95	7.85	48.28	0.87	0.79	0.93
7.11	7.05	48.43	0.70	0.65	0.74
4.00	5.83	48.51	0.61	0.57	0.68
4.86	4.57	48.70	0.51	0.44	0.55
7.09	4.31	49.07	0.72	0.67	0.78
7.28	4.55	49.13	0.76	0.70	0.83
8.22	8.91	50.02	0.94	0.84	0.99
5.72	4.72	50.26	0.85	0.77	0.91
4.89	9.73	50.75	0.75	0.65	0.79
6.09	5.92	51.05	0.67	0.57	0.74
7.49	7.77	51.08	0.83	0.73	0.91
6.61	8.37	51.92	0.86	0.77	0.90
9.06	6.61	52.61	0.82	0.78	0.87
8.53	9.46	52.74	0.87	0.78	0.94
6.10	6.32	52.89	0.91	0.83	0.95

9.29	9.93	52.97	1.00	0.92	1.06
1.71	10.23	53.69	0.84	0.76	0.89
8.89	7.26	53.89	0.94	0.83	1.01
9.00	9.51	53.90	0.90	0.84	0.96
7.75	9.33	55.74	0.92	0.85	0.99
6.82	6.65	55.85	0.67	0.60	0.72
9.51	9.64	55.96	1.07	0.93	1.11
4.21	9.90	56.31	0.84	0.75	0.90
12.49	11.05	57.54	0.82	0.76	0.86
9.39	10.25	57.54	0.85	0.75	0.89
10.50	10.12	57.61	1.09	0.99	1.15
5.26	7.53	57.88	0.79	0.74	0.84
9.17	6.40	57.96	0.94	0.85	1.00
8.82	9.32	58.30	0.96	0.87	1.00
8.87	5.89	58.35	0.94	0.86	1.02
8.92	8.77	58.72	0.91	0.84	0.93
7.85	7.59	58.86	0.83	0.76	0.88
8.67	9.04	58.91	0.92	0.86	0.97
4.97	6.50	59.12	0.94	0.87	1.00
4.49	4.80	59.13	0.45	0.41	0.48
9.75	10.13	59.46	1.08	0.93	1.13
9.45	9.63	59.59	1.01	0.91	1.06
9.17	8.83	60.12	0.99	0.84	1.04
8.04	9.93	60.56	1.01	0.94	1.09
12.24	10.21	60.65	1.02	0.95	1.06
6.44	6.62	62.10	0.69	0.60	0.77
6.55	6.60	62.27	0.76	0.64	0.84
9.82	12.53	64.05	1.35	1.25	1.37
10.36	10.20	65.37	1.11	0.96	1.15
10.25	10.18	66.48	1.06	0.96	1.09

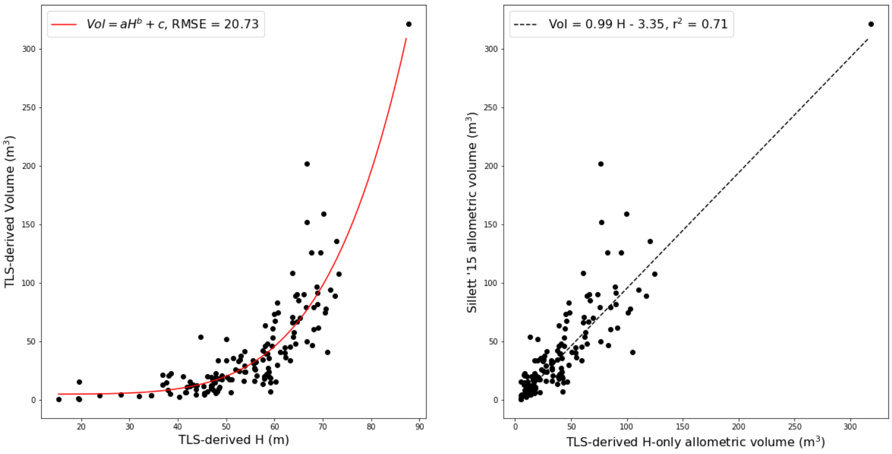

Figure S1: Volume estimated from TLS-derived values of H only, plotted in arithmetic space (left panel); scatter of volume predicted from TLS-derived H only allometry, against volume predicted by Sillett et al. (2015)[19] allometry (right panel).
